# Outcome of community- versus hospital-acquired intra-abdominal infections in intensive care unit: a retrospective study

**DOI:** 10.1186/s12871-020-01209-1

**Published:** 2020-12-01

**Authors:** Timothée Abaziou, Fanny Vardon-Bounes, Jean-Marie Conil, Antoine Rouget, Stéphanie Ruiz, Marion Grare, Olivier Fourcade, Bertrand Suc, Marc Leone, Vincent Minville, Bernard Georges

**Affiliations:** 1 Département D’Anesthésie-Réanimation (Department of Anesthesia and ICU), CHU Rangueil (University Hospital Centre of Rangeuil), 1 Avenue du Professeur Jean Poulhes TSA 50032, 31059 Toulouse, France; 2 Laboratoire de Bactériologie et Hygiène (Bacteriology and Hygiene Laboratory), Institut Fédératif de Biologie (Federative Institute of Biology), 330 Avenue de Grande Bretagne, Cedex 9, 31059 Toulouse, France; 3 Service de Chirurgie Digestive (Department of Gastrointestinal Surgery), CHU Rangueil (University Hospital Centre of Rangueil), 1 Avenue du Professeur Jean Poulhes, 31059 Toulouse, France; 4 Aix Marseille Université, Assistance Publique Hôpitaux de Marseille (Public Hospitals of Marseille), Service D’Anesthésie-Réanimation (Department of Anaesthesia and ICU), Hôpital Nord, Chemin des Bourrely, 13015 Marseille, France

**Keywords:** Intra-abdominal infection, Peritonitis, Outcome, Microbiology, Intensive care unit

## Abstract

**Background:**

To compare patients hospitalised in the intensive care unit (ICU) after surgery for community-acquired intra-abdominal infection (CA-IAI) and hospital-acquired intra-abdominal infection (HA-IAI) in terms of mortality, severity and complications.

**Methods:**

Retrospective study including all patients admitted to 2 ICUs within 48 h of undergoing surgery for peritonitis.

**Results:**

Two hundred twenty-six patients were enrolled during the study period. Patients with CA-IAI had an increased 28-day mortality rate compared to those with HA-IAI (30% vs 15%, respectively (*p* = 0.009)). At 90 days, the mortality rates were 36.7 and 37.5% in the CA-IAI group and HA-IAI group, respectively, with a similar APACHE II score on admission (median: 21 [15–25] vs. 21 [15–24] respectively, *p* = 0.63). The patients with HA-IAI had prolonged ICU and hospital stays (median: 17 [7–36] vs. 6[3–12] days, *p* < 0.001 and 41 [24–66] vs. 17 [7–32] days, *p* = 0.001), and experienced more complications (reoperation and reintubation) than those with CA-IAI.

**Conclusion:**

CA-IAI group had higher 28-day mortality rate than HA-IAI group. Mortality was similar at 90 days but those with HA-IAI had a prolonged ICU and hospital stay. In addition, they developed more complications.

## Background

In some studies, the mortality of patients developing severe intra-abdominal infection (IAI) reaches 50% [1–3]. Among severe intra-abdominal infections, peritonitis is classified according to one of 3 categories: primary, with a medical aetiology and treatment; secondary, of surgical origin representing the most prevalent cases; and tertiary, with an ongoing intra-abdominal infection despite appropriate care [2] . In the case of secondary peritonitis, treatment is surgical, requiring peritoneal washing after bacteriological sampling, and repair of gut lesions, associated with antibiotics and support for organ failure [2, 4]. Two types of IAI are defined: community-acquired IAI (CA-IAI) and hospital-acquired (HA-IAI) [3].

CA-IAI has a florid presentation, with fever and peritoneal signs. *Escherichia coli* (*E. coli*) is the most frequently found bacteria [5–7].

In contrast, peritoneal signs are less apparent in patients with HA-IAI. Although *E. coli* is still the most frequent bacteria, antimicrobial resistance is commonplace. *Pseudomonas aeruginosa*, extended spectrum beta lactamase *Enterobacteriae or* methicillin-resistant *Staphylococcus aureus* are also involved, depending on the local ecology [5, 8–10].

The aim of this study was to compare the 28-day mortality rate between patients admitted to ICU with CA-IAI and HA-IAI. Secondary objectives are to describe mortality-related factors, complications, length of stay, microbiological findings and antibiotic treatment.

## Methods

This was a retrospective study enrolling ICU patients from two university hospitals from January 2009 to May 2013, treated for secondary or tertiary peritonitis. The local ethics committee (Comité d’Ethique de la Recherche de Toulouse) approved this study (No. 61–1112). According to French legislation, patient consent was waived.

The patients treated for secondary IAI with no surgical treatment (radiological puncture or withdrawal of care), or patients transferred to ICU 48 h after surgical procedure were not included. The patients were treated according to local and international guidelines [2, 4].

### Definitions

We defined two groups of patients, CA-IAI group and HA-IAI group, according to national and international guidelines [11, 12]. The HA-IAI group comprised patients with postoperative IAI and IAI diagnosed at least 48 h after hospitalisation, regardless of the reason for admission. Patients were classified in the CA-IAI group if they didn’t meet HA IAI definition. The attending physician diagnosed postoperative IAI, but we included only patients requiring a surgical procedure.

### Surgical management

All included patients underwent surgery and required laparotomy. Laparotomy was decided by the attending surgeon, and justified by severity of the infection and/or because it was a postoperative IAI. The attending surgeon confirmed the intra-abdominal infection, performed peritoneal lavage with isotonic sodium chloride solution after peritoneal sample. Surgical repair and/or resection were achieved as the attending surgeon decided, and ostomies were preferred at primary anastomosis. Temporary abdominal closure with negative pressure was not routinely used and left at the attending surgeon’s discretion. Patients were reoperate on-demand in most cases, except for patients with mesenteric ischemia who were reoperate 48 h after the initial surgery.

### Data collected

We recorded baseline demographic data [age, gender, body mass index (BMI)], medical history, the use of antibiotic treatment in the 28 days prior to surgery, lesion site, the type of IAI (localised or generalised), the Mannheim Peritonitis Index and the APACHE II score on admission to the ICU [13, 14]. During the first 24 h after surgery, we recorded the need for mechanical ventilation for more than 24 h, the need for norepinephrine infusion, plasma creatinine concentrations above 150 μmol/L, prothrombin times below 50% and platelet counts of less than 50,000/mm^3^. In the 48 h after surgery, the need for renal replacement therapy was also documented.

Peritoneal sample cultures with the antibiotic susceptibility test, the empirical antibiotic treatment chosen and suitability in relation to the bacterial results obtained were recorded. Microbiological procedures were those routinely used in the local laboratory, according to the French Society of Microbiology [15].

We assessed the appropriateness of the empirical antimicrobial therapy, defined by at least one antimicrobial active against the pathogens that were identified by the microbiological cultures. We defined empiric antibiotic treatment as antibiotic given before bacteriological results, and directed as directed by antibiotic susceptibility.

The hospital and ICU length of stay from hospital admission (ward or ICU), 28-day and 90-day mortality rate after surgery, 28 ventilator-free days and 28 antibiotic-free days over the postoperative period, re-intubation, limitation or withdrawal of care and revision surgery were also documented.

### Statistical analysis

Statistical analyses were performed with R software (R Core Team (2014). R: A language and environment for statistical computing. R Foundation for Statistical Computing, Vienna, Austria. URL http://www.R-project.org/). Student’s t test or Mann-Whitney’s test were used to compare quantitative variables as appropriate, and Chi2 test or Fisher test to compare binomial variables, as required. Data was expressed as median values with 1st and 3rd interquartile or percentage. We used Kaplan-Meier curves to represent changes in the mortality rate in the first 28 days post-admission. We carried out Cox model regression in order to establish mortality-related factors. We included significant variables in the univariate analysis when this variable was present on or before admission. We chose the model with the higher concordance index. Before modelling, we used multiple imputations to deal with missing data. Categorical variables are expressed as number (%). Quantitative variables are expressed as median [1st – 3rd quartile]. A two-sided *p* value of less than 0.05 was considered to be statistically significant.

## Results

**Fig. 1:**
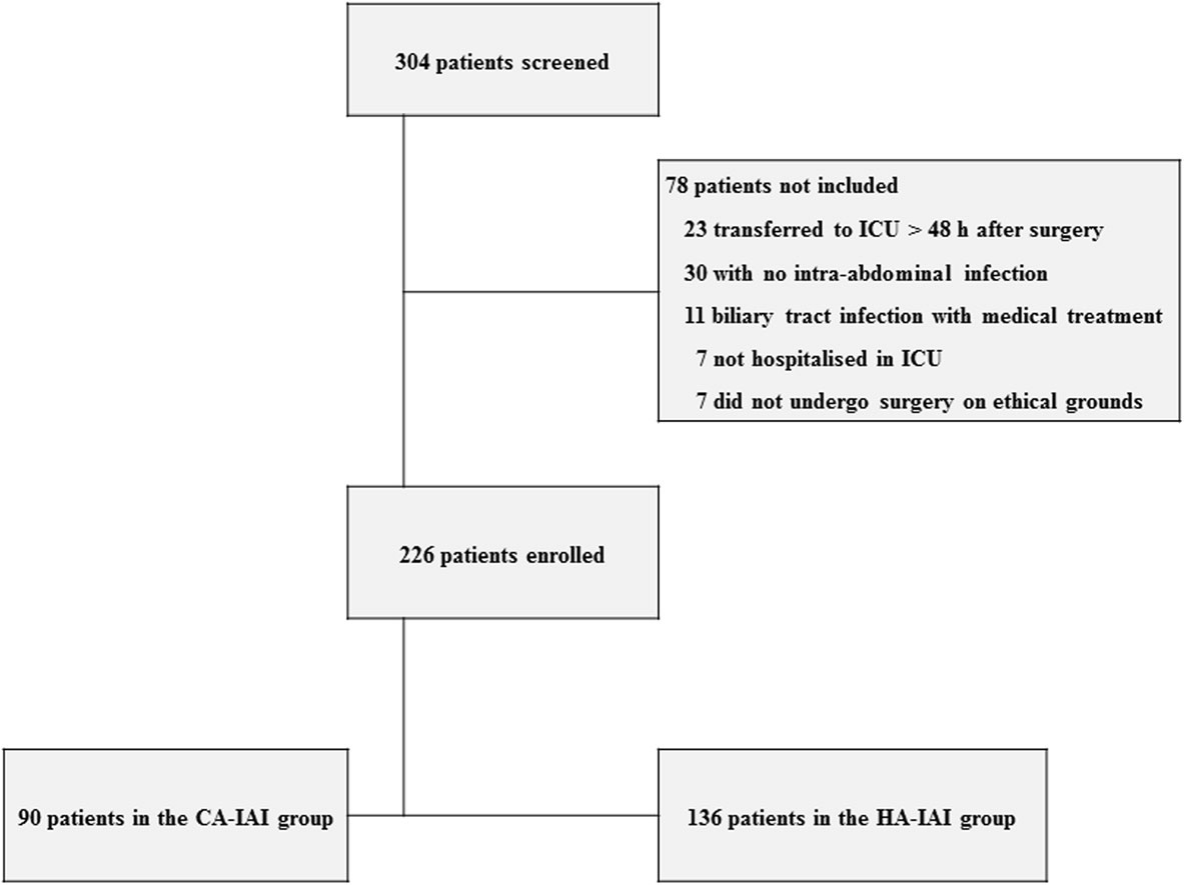
Flow chart of enrolment. CA-IAI: community-acquired Intraabdominal Infection; HA-IAI: hospital-acquired Intraabdominal Infection

During the study period, 304 cases were screened, 78 were excluded and 226 were enrolled - 90 in the CA-IAI group and 136 in the HA-IAI group (Fig. 1). Demographic characteristics are summarised in Table 1. The 2 groups had similar baseline characteristics except for prior antibiotics administration in the 28 days before surgery (16% in the CA-IAI group vs. 52% in the HA-IAI group, *p* < 0.001) and aetiology.

**Table 1:** Demographic characteristics of the study population

	CA-IAI		HA-IAI		*p*
	(*n* = 90)		(*n* = 136)		
Male, n (%)	52	(57.8)	85	(62.5)	0.48
Age, years median [IQR]	66	[52–78]	67	[57–76]	0.86
BMI median [IQR]	24	[22–30]	25	[22–28]	0.84
Medical history, n (%)					
coronary disease	25	(27.8)	29	(21.3)	0.26
arterial occlusive disease	16	(17.8)	17	(12.5)	0.36
cardiac insufficiency	5	(5.6)	6	(4.4)	0.26
chronic renal failure	5	(5.6)	11	(8.1)	0.64
chronic dialysis	1	(1.1)	1	(0.7)	1
cirrhosis	4	(4.4)	6	(4.4)	1
diabetes mellitus	16	(17.8)	19	(13.9)	0.44
immunodeficiency	16	(17.8)	23	(16.9)	0.86
abdominal surgery	39	(43.3)	64	(47.1)	0.58
Prior antibiotic treatment, n (%)	14	(15.6)	70	(51.5)	< 0.001
Generalised peritonitis, n (%)	28	(31.1)	56	(41.2)	0.62
Localisation, n (%)					0.36
colon	38	(42.2)	61	(44.9)	
small intestine	28	(31.1)	28	(20.6)	
stomach/duodenum	15	(16.7)	20	(14.7)	
other	9	(10)	25	(18.4)	
Aetiology					< 0.001
Perforation	59	(65.6)	34	(24.5)	
Ischaemia	20	(22.2)	19	(13.7)	
Anastomotic leakage	0	(0)	35	(25.2)	
Po with no lesion found	0	(0)	28	(20.6)	
Po abscess	0	(0)	6	(4.3)	
Trauma	8	(8.9)	5	(3.6)	
Other	3	(3.4)	9	(6.5)	
APACHE II	21	[15–25]	21	[15–24]	0.63
MPI	23	[16–28]	24	[17–28]	0.24
Mechanical ventilation > 24 h, n (%)	61	(67.8)	107	(78.7)	0.052
Norepinephrine, n (%)	63	(70.0)	106	(78.0)	0.12
Plasma creatinine level > 150 μmol/L, n (%)	41	(45.6)	63	(46.3)	0.89
PT < 50%, n (%)	23	(25.6)	38	(28.0)	0.66
Platelet < 50,000 /mm^3^, n (%)	16	(17.8)	15	(11.0)	0.16
Renal replacement therapy, n (%)	25	(27.8)	50	(36.8)	0.15

*BMI* Body mass index. Immunodeficiency was defined by haematological cancer, active solid cancer, AIDS Immunosuppressive therapy and corticosteroid therapy initiated at least 1 month before. MV > 24 h: Mechanical ventilation still ongoing 24 h after admission. *PT* Prothrombin time. *APACHE II* APACHE II score on admission after surgical management. *MPI* Mannheim Peritonitis Index. Po: postoperative

### 28-day mortality rate and mortality-related factors

**Fig. 2:**
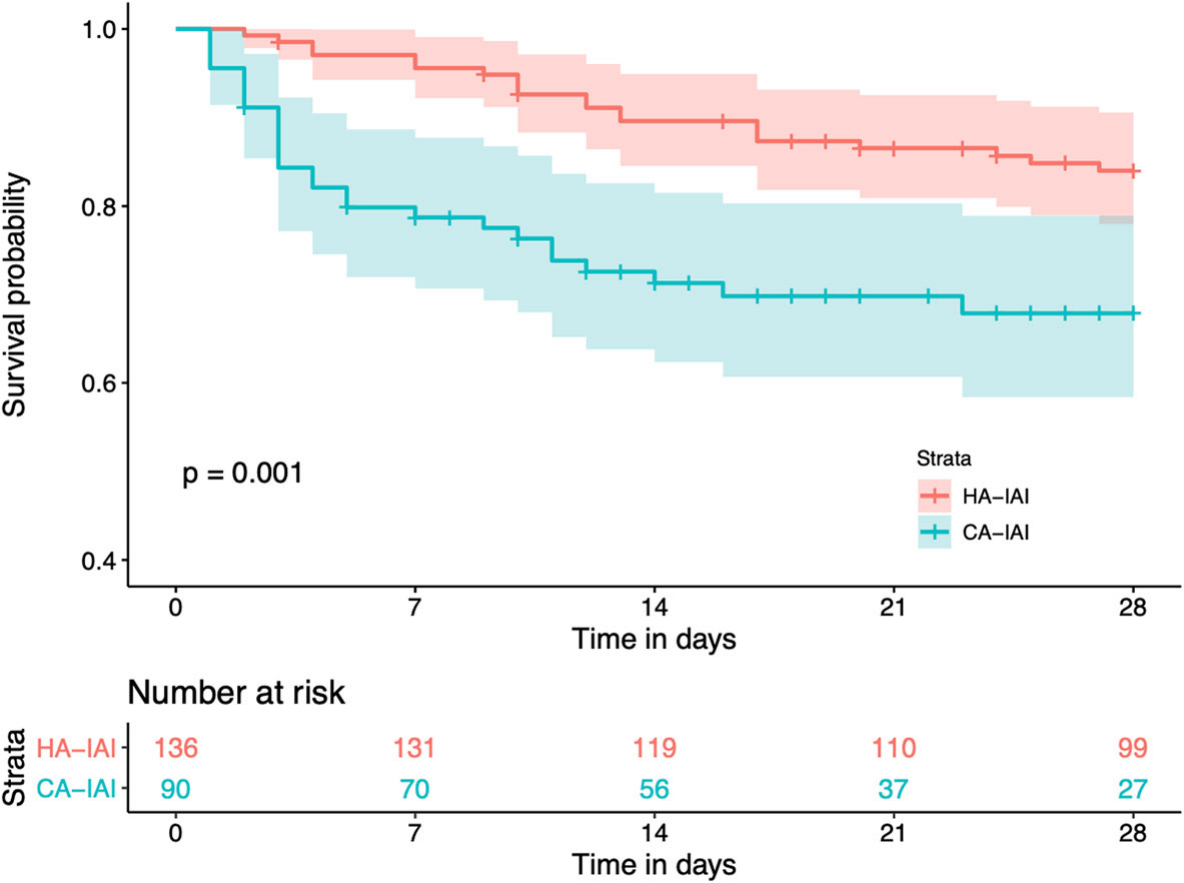
Kaplan-Meier curve of survival rate at 28 days, with 95% confidence interval. Blue line: CA-IAI: community-acquired Intraabdominal Infection; Red line: HA-IAI: hospital-acquired Intraabdominal Infection

**Fig. 3:**
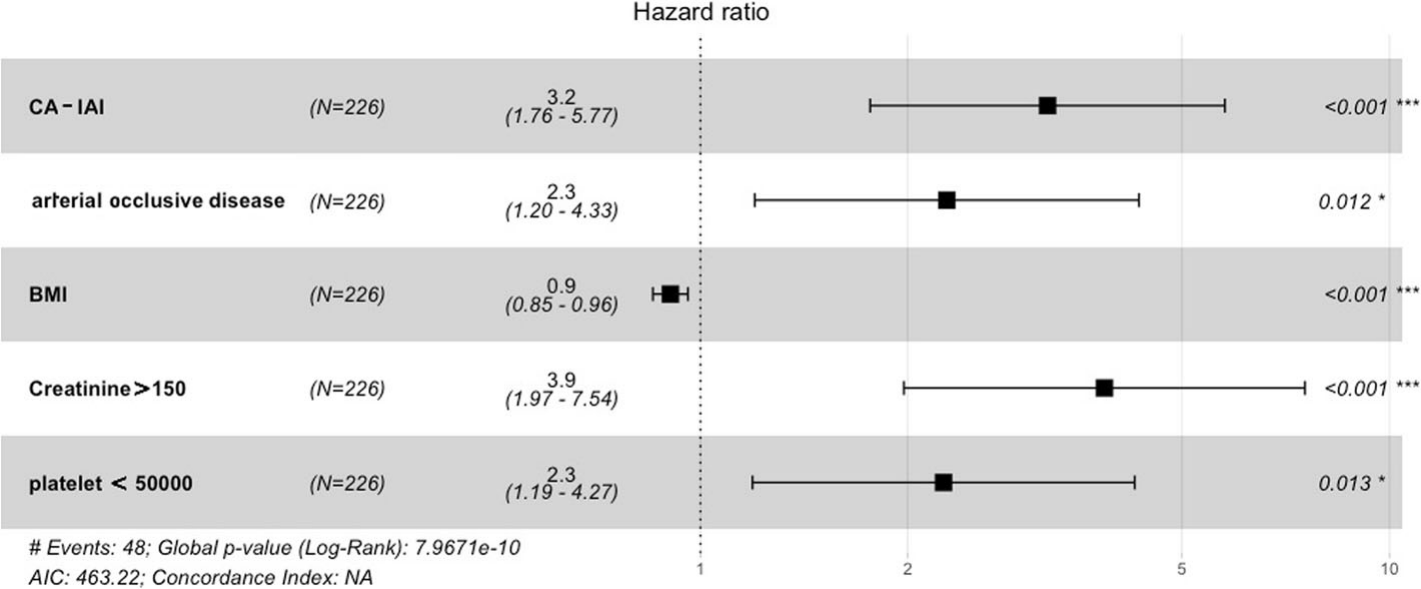
Cox proportional hazard model. CA-IAI: community-acquired Intraabdominal Infection; BMI: Body mass index superior to 23; platelet < 50,000: platelet count inferior to 50,000/mm^3^; Creatinine > 150: serum creatinine rate superior to 150 μmol/l

The 28-day mortality rate was 30 and 15% in the CA-IAI group and the HA-IAI group, respectively (*p* = 0.009). The Kaplan-Meier curve analysis confirmed this difference (*p* = 0.001) (Fig. 2). Using a Cox model, CA-IAI (hazard ratio (HR): 3.0 [1.7–5.5], *p* < 0.001), peripheral vascular disease history (HR: 2.10 [1.07–3.99], *p* = 0.031), platelet count below 50,000 /mm^3^ (HR: 1.9 [1.01–3.73], *p* = 0.047), plasma creatinine above 150 μmol/L (HR: 3.00 [1.43–6.13], *p* = 0.003) were associated with the 28-day mortality rate, and BMI above 23 (HR: 0.91 [0.86–0.97], *p* = 0.003) was associated with a lower rate (Fig. 3). The concordance index was 0.77. The likelihood ratio test, the Wald test and the log-rank test were significant (*p* < 0.001).

### Other outcomes

Compared to the CA-IAI group, the HA-IAI group had a prolonged ICU and hospital stay (17 [7–36] vs. 6 [3–12] days, *p < 0.001* and 41 [24–66] vs. 17 [7–32 days, *p* < 0.001, respectively) (Table 2). This group required more repeat operations (21% vs. 9%) and re-intubation (41% vs. 17%). A decision to withdraw care was taken for 18% of patients in the CA-IAI group and 19% of patients in the HA-IAI group (*p* = 0.85). The 90-day mortality rate was similar in both groups (35.6 vs. 31.6 for CA-IAI group and HA-IAI group, respectively, *p* = 0.54). On day 28, the number of mechanical ventilation-free days was 20 [0–27] and 11 [0–23] days in the CA-IAI group and the HA-IAI group, respectively (*p* = 0.039). Similarly, the number of antibiotic-free days was 13 [0–18] and 5 [0–13] days in the CA-IAI group and the HA-IAI group, respectively (*p* = 0.024).

**Table 2:** Length of stay and complications

	CA-IAI (*n* = 90)	HA-IAI (*n* = 136)	*p*
ICU LOS, median [IQR]	6 [3–12]	17 [7–36]	< 0.001
Hospital LOS, median [IQR]	17 [7–32]	41 [24–66]	< 0.001
Reoperation, n (%)	8 (9.0)	28 (20.7)	0.019
Reintubation, n (%)	17 (19.5)	54 (40.6)	< 0.001
Withdrawal of care, n (%)	16 (18.4)	26 (19.4)	0.85
Death at 90 days, n (%)	32 (35.6)	43 (31.6)	0.54

*ICU* Intensive care unit; *LOS* Length of stay; *IQR* Interquartile range

### Bacteriological findings and antibiotic treatment

In our study, peritoneal samples were collected from 49 (54%) patients in the CA-IAI group and 119 (88%) patients in the HA-IAI group (Table 3), resulting in 102 and 257 isolates in the CA-IAI and HA-IAI groups, respectively. In both groups, *E. coli* was the main bacteria identified, and *Enterococcus faecalis* was the main Gram-positive cocci found.

**Table 3:** Main bacteriological findings in CA-IAI (46 patients) and HA-IAI groups (111 patients)

	CA-IAI group		HA-IAI group	
	N isolate	%	N isolate	%
Gram – aerobes	37	36.3	116	45.1
*Escherichia coli*	23	22.5	56	21.8
*Klebsiella* spp	8	7.8	17	6.6
*Citrobacter* spp	2	2.0	3	1.2
*Proteus* spp	1	1.0	10	3.9
*Enterobacte*r spp	1	1.0	12	4.7
*Pseudomonas aeruginosa*	1	1.0	11	4.3
Others	1	1.0	7	2.7
Gram + aerobes	57	55.9	115	44.7
*Enterococcus faecalis*	12	11.8	30	11.7
*Enterococcus faecium*	11	10.8	25	9.7
*Enterococcus* Other	7	6.9	12	4.7
*Streptococcus* spp	12	11.8	17	6.6
*Staphylococcus aureus*	1	1.0	7	2.7
*Coagulase-negative Staphylococcus*	9	8.8	16	6.2
Others	5	4.9	7	2.7
Anaerobic	9	8.8	25	9.7
*Bacteroides* spp	2	2.0	11	4.3
Others	7	6.9	14	5.4
Yeast				
*Candida*				
*Albicans*	10	55.6	25	58.1
*Glabrata*	0	0.0	6	14.0
*Parapsilosis*	3	16.7	1	2.3
*other Candida*	2	11.1	8	18.6
Others	3	16.7	3	7.0

In both group, *Candida albicans* was the main yeast identified, 10 cases in CA-IAI group vs. 25 in the HA-IAI (*p* = 0.85). Other species were found in 8 cases in the CA-IAI group (3 *C. parapsilosis*, 1 *C. inconspicua*, 1 *C. norengensis*, 1 *Saccharomyces cerevisae*, 1 *Sporopachydermia lactativora* and 1 unidentified yeast), and in 18 cases in the HA-IAI group (6 *C. glabrata*, 4 *C. krusei*, 2 *C. tropicalis*, 1 *C. kefir*, 1 *C. fumata*, 1 *C. inconspicua*, 1 *Aspergillus fumigatus*, 1 *Saccharomyces cerevisae*, and 1 unidentified yeast). For patients with positive peritoneal samples (46 samples in CA-IAI group, 111 in the HA-IAI group), the antibiotic treatment was adequate for 38 patients in the CA-IAI group (78%) and for 93 patients in the HA-IAI group (84%) (*p* = 0.4). Inappropriate antibiotic treatment was due to the presence of *E. faecium* (3 vs. 10 patients in the CA-IAI and HA-IAI groups, respectively). The antibiotic treatment was subsequently adjusted according to antibiotic susceptibility in 73% of patients in the CA-IAI group and 82% in the HA-IAI group (*p* = 0.15). A combination of piperacillin/tazobactam and amikacin was the most widely prescribed antibiotic in the 2 groups (43 and 63% in the CA-IAI and HA-IAI groups, respectively, *p* = 0.003). An antifungal treatment was administered concomitantly for 40 patients in the CA-IAI group (44%, caspofungin in 21 cases, fluconazole in 19 cases) and 78 patients in the HA-IAI group (57%, caspofungin in 49 cases, fluconazole in 30, and voriconazole in 1 case) (*p* = 0.057).

## Discussion

In our study, CA-IAI patients had a higher 28-day mortality rate than those with HA-IAI. However, at 90 days, the mortality rates were similar in both groups. Based on our knowledge, few studies actually compare outcomes for patients with CA-IAI and HA-IAI. Van Ruler et al. noted mortality rates of 13% for patients with CA-IAI and 30% for those with HA-IAI, including patients with an APACHE II score above 10 [7]. Montravers et al. found a mortality rate of 4% for patients with CA-IAI and 12% for patients with hospital-acquired, non-postoperative peritonitis in a mixed population of ICU and non-ICU patients [16]. Inui et al. observed a mortality rate of 3.8% for patients with CA-IAI and 8.4% for HA-IAI patients. This study included IAI with or without surgical treatment [17]. In a multicentre study, no significant difference in mortality rate was reported in patients with CA-IAI and HA-IAI [5]. These findings probably reflect differences in the inclusion criteria, endpoint definitions and the type of IAI.

We can only assume the reason for the difference in the 28-day mortality rate. Our two groups are similar in terms of severity criteria and APACHE II score. However, delay between the onset of symptoms, initiation of antibiotic treatment and surgical management could not be reliably collected, which can be a major confounding bias. Time between clinical onset and antibiotics or operating room for patients in CA-IAI group could have been more important than recommended, which could explain the increased mortality rate. No peritoneal sample was collected for a large number of CA-IAI patients. Therefore, we did not know whether antimicrobial treatment was adequate for these patients. Furthermore, in-patients were more likely to receive broad-spectrum antibiotics. Some patients from the HA-IAI group were already in the ICU when peritonitis developed, and therefore returned to the ICU after surgical management regardless of the severity criteria. These differences could explain why death occurred earlier in the CA-IAI group than in the HA-IAI group. However, it should be noted that the mortality rate was similar at 90 days.

It is important to notice that the 2 ICUs from where patients were included take care of the most severe cases hospitalized in our institution. Less severe cases, with only one organ failure and not mechanically ventilated are usually hospitalized in other intensive care units.

A medical history of arterial occlusive disease, platelet count below 50,000/mm3, creatinine serum levels greater than 150 μmol/l, and a high APACHE II score were also associated with a worse outcome. A BMI of over 23 was associated with a better outcome. Thrombocytopenia had already been described as a mortality-related factor in IAI, and acute kidney injury in critically ill patients and sepsis in particular [18, 19]. A meta-analysis studying overweight, obesity and sepsis reported an association with a better outcome [20]. To the best of our knowledge, arterial occlusive disease has not been previously described as a mortality-related factor, but its association with coronary disease is well known, which could explain our findings [21].

Other outcomes, as defined by our study, have generally been poorly reported in previous studies, except for reoperation. This last endpoint is generally higher in HA-IAI patients [9, 17]. As in other studies, we report longer ICU and hospital stays for HA-IAI patients compared to those with CA-IAI [17, 22].

The bacteriological findings were consistent with the literature, except for the rate of anaerobic bacteria [5, 23]. This may be attributed to poor quality of sampling, conditioning or logistics of the peritoneal sample. Our institution has taken measures to improve this. Empirical antibiotic therapy was appropriate in 72.5% of the CA-IAI group and 82.2% of the HA-IAI group. The presence of amoxicillin-resistant *Enterococcus faecium* was the main reason for inappropriate antibiotic therapy, as confirmed in earlier findings [5, 24, 25]. A combination of piperacillin/tazobactam with amikacin was the most widely prescribed empirical antibiotic therapy. It was administered to approximately 50 % of patients. For CA-IAI patients, this treatment is in accordance with French and International guidelines, although the benefit of aminoglycosides is not proven in this indication [2, 4, 26, 27]. As regards HA-IAI patients, carbapenems are currently proposed in guidelines when specific conditions are found [2, 4]. Otherwise, piperacillin/tazobactam is indicated, possibly in conjunction with an aminoglycoside and/or vancomycin. Inadequate empirical antibiotic treatment is associated with poor prognosis, increased morbidity and mortality rates, reoperation and prolonged ICU or hospital stays [22, 28–30].

Our study has several limitations. Firstly, this is a retrospective study with missing data. Especially as already mentioned, time between diagnosis, antibiotics and surgery were not consistently or reliably recorded. These parameters are known to have a major impact on patients’ outcome, and the lack of these data might affect our results. Antibiotic treatment duration for IAI was not always explicitly reported, and knowing when the course stopped and a new one for other infection begin was not always possible, explaining why we used 28-antibiotics free days. If focus control of the infection was possible after surgery was also not clearly reported, but we did not found any evidence to the contrary. Secondly, the patients were included from two hospitals only, making it difficult to extrapolate our findings. Thirdly, not all patients had a peritoneal sample prior to surgery, particularly in the CA-IAI group. As mentioned above, the impact of the initial antibiotic on the microbiological findings, which is generally associated with good outcome in terms of mortality rates or complications, was not analysed in our study. This situation had been already reported in another study, and the rate of peritoneal sampling needs to be improved as recommended in current guidelines [24, 31, 32]. Fourthly, recruitment period begun in 2009, and critically ill patients management has evolved since then, which could make extrapolation of our results difficult. And lastly, we did not included patients transferred 48 h after surgery as we assumed their transfer were not directly related from IAI and septic shock, or if so, would have been done after revision, and were more related to patients’ medical history. This might lead to selection bias and less daily practice representability.

## Conclusion

In our study, CA-IAI patients were surprisingly at higher risk of 28-day mortality after ICU admission than those with HA-IAI. The need for reoperation and reintubation increased in the HA-IAI group in conjunction with prolonged ICU and hospital stays. However, the increased 28-day mortality rate was not confirmed at 90 days. Therefore, long-term outcomes should be assessed.

## Data Availability

The datasets used and analysed during the current study are available from the corresponding author on reasonable request.

## Abbreviations

IAI: Intra-abdominal infection
CA-IAI: Community-acquired intra-abdominal infection
HA-IAI: Hospital-acquired intra-abdominal infection
ICU: Intensive care unit
